# Utilization of FMOC-3F-PHE hydrogel for encapsulation of *Zanthoxylum armatum* and *Cinnamomum camphora* oil for enhancing their antibacterial activity

**DOI:** 10.1186/s13104-022-06163-4

**Published:** 2022-08-12

**Authors:** Nasla Shakya, S. Budha Chettri, Susan Joshi, Annada Rajbhandary

**Affiliations:** 1 Research Institute for Bioscience and Biotechnology (RIBB), Kathmandu, Nepal; 2 Tri-Chandra Multiple Campus, Durbar Marga, Kathmandu, Nepal

**Keywords:** Hydrogel, Fmoc-3F-Phe amino acid, Essential oil, *Zanthoxylum armatum*, *Cinnamomum camphora*, Antibacterial, Sustained-release

## Abstract

**Objective:**

While essential oils have many applications in medicine, not many studies have been done in the past to address issues of active targeting, enhancing bioavailability and reducing toxicity at higher concentrations. Herein, we used Fmoc-3F-Phe amino acid hydrogels to address such issues by encapsulating essential oils, *Zanthoxylum armatum* and *Cinnamomum camphora*, in its system and allowing sustained-release of these oils onto bacterial assays of *E. coli* ATCC 25922*, P. hauseri* NBRC 3851*, M. luteus* KACC 13377, and *B. subtilis* ATCC 66333 for probing enhanced antibacterial properties of the oils by prolonging its efficacy through controlled-release mechanism.

**Results:**

We found that while *Zanthoxylum* oil showed no particular difference in enhancing the antibacterial property against the three fast growing bacteria, however profound variation was observed against slow growing bacteria *B. subtilis.* The hydrogel encapsulated oil was able to retain its antibacterial property for a longer time while directly applied oil could not for this bacteria. Even for highly volatile camphor oil, the oil itself failed to show any antibacterial property with direct use, however the hydrogel encapsulated oil was able to show excellent antibacterial property for *B. subtilis* and *M. luteus* through prohibition of sublimation via encapsulation.

**Supplementary Information:**

The online version contains supplementary material available at 10.1186/s13104-022-06163-4.

## Introduction

Medicinal plants have been used as primary sources of medicine since time unknown. Researches now have revealed that these types of medicinal plants are rich sources of various metabolites that possess many therapeutic properties [1–3]. Moreover, medicinal plants are gaining much attention in recent years due to various advantages over conventional drugs such as these possessing lesser side effects and even being more biocompatible [4]. While the medicinal uses of medicinal plant derived essential oils (EO) is profound, the therapeutic efficacy of these oils still seems limited due to lack of targeting capacity, less specificity, toxicity at higher concentrations, poor bioavailability and reduced efficiency of absorption caused by sublimation of the oils [1, 4]. Here, modern drug delivery system such as hydrogels pose as alternative route to resolve these key issues which have been rarely used to formulate with EOs to solve such problems [2–5].

Hydrogels, which are semi-solid materials with three-dimensional network of polymers have both solid and liquid like properties and have many commercial applications and biomedical uses [6, 7]. While, most self-assembled hydrogels reported are made from biologically inspired polymers, low molecular weight (LMW) non-polymeric self-assembling hydrogels are gaining more popularity due to its better biocompatiblity, biodegradability and the presence of weaker non-covalent forces that allow formation of softer and tunable gels [8]. Fmoc-Phe amino acid based hydrogels fall under this category of hydrogelators that have been extensively studied in the recent years owing to the ease of hydrogelation and its numerous applications [9]. For the purposes of our study, we used Fmoc-3F-Phe hydrogelator that have been utilized in the past to form homogenous and rigid gels and are well known for its rapid formation of gelation network in normal room temperature [10]. These hydrogels were utilized to formulate it with EOs, *Zanthoxylum armatum* (sichuan pepper) and *Cinnamomum camphora* (camphor) which have uses in cuisines, commercial and homeopathic applications [11–14]. Among the two EOs utilized, camphor oil is especially known for being highly volatile and unstorable [14–16]. We used the gels for incorporation of these two EOs and deposited these onto the bacterial surfaces to study the applications of hydrogels as effective delivery system of EOs to enhance the bactericidal effects of the oils.

## Main text

### Results and discussion

**Fig. 1:**
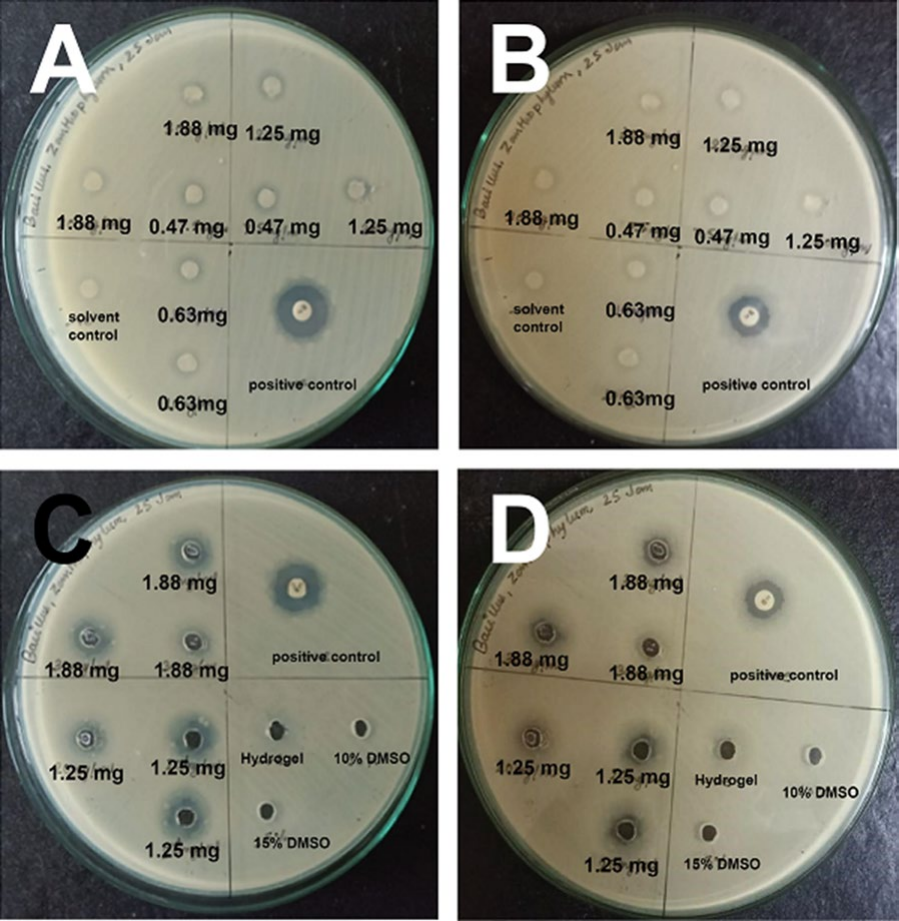
Antibacterial Assay of *B. subtilis* at various amounts (0.47 mg, 0.63 mg, 1.25 mg and 1.88 mg) of *Zanthoxylum* oil applied in paper discs at incubation times (**A** at 14 h, **B** at 22 h); and at 1.25 mg and 1.88 mg of *Zanthoxylum* oil encapsulated in Fmoc-3F-Phe hydrogels at incubation times (**C** at 14 h and **D** at 22 h)

EOs of the seeds of the Sichuan pepper and leaves of the Camphor plants were extracted via hydrodistillation (Additional file 1: 1.2). These were then encapsulated in the Fmoc-3F-Phe hydrogels to form Gel-EOs to study antibacterial efficiency of the oils after slowly releasing through the gels after being placed on the bacterial assays of *E. coli (EC)*, *P. hauseri (PH)*, *M. luteus (MH)* and *B. subtilis (BS)* bacteria. For the experiments, firstly, antibacterial assay were prepared to identity zone of inhibition (ZOI) resulting from direct application of the oils only, at low and high concentrations dissolved in isopropanol via disc diffusion method (Additional file 1: 1.6 and 1.7). In ideal conditions of slow-release mechanism, the gels should be able to sustain high concentration of the EO within itself and then allow slow-release of the oils from its system to ensure consistent amounts of doses over a long period for improved efficacy. Therefore, the antibacterial assay experiment was also repeated by formation of Gel-EOs at high concentrations using the well method (Additional file 1: 1.8). From the results obtained, it can be deduced that *Zanthoxylum* oil can show measurable ZOI (~ 9 mm) using mass as low as 0.47 mg for all four bacteria tested (Fig. 1A, B and Additional file 1: Fig. S1A–C, Table S1; Table 1). While the solvent used to dissolve the oil, isopropanol itself is toxic to the bacteria which is why the solvent control also shows inhibitory activities, the ZOI of the EOs at 0.47 mg is higher (compare 9 mm vs. 7 mm) indicating that the additional inhibition in the bacteria was caused by the oil placed on the assay. With higher amount of EO at mass 0.63, 1.25 and 1.88 mg, the ZOI increased accordingly (Additional file 1: Table S1A, B). With ZOI seen at amount as low as 0.47 mg for all four bacteria which is a very low amount to show potency against the bacteria, for the purposes of this study, amount much higher than 0.47 mg was used to form Gel-EOs to allow slow-release of the oils and probe improved or prolonged efficiency. However, it can be observed that the ZOI was only slightly higher for experiments with *Zanthoxylum* Gel-EO, as opposed to the use of oil directly when the same amount of oil was used for bacteria *EC*, *PH* and *ML* (compare Additional file 1: Table S1A, B; Fig. S1).The incubation time for three bacteria was kept at 14 h and a follow up reading at 22 h did not show drastic difference in the ZOI (Additional file 1: Fig. S1). Therefore it can be deduced that for these three types of bacteria, that the difference between the use of oil directly on to the assay and those from Gel-EOs is minimal and do not show major differences although results obtained with the gels were slightly improved. However, significant difference could be observed in case of *BS* where, the EO applied directly showed good amount of ZOI at 14 h of incubation but the ZOI had almost entirely disappeared by the 22 h time (Fig. 1, Table 1). By comparison, the Gel-EO showed comparable amount of ZOI at 14 h which remained substantial even until 22 h with ZOI only slightly diminishing (compare Fig. 1A, B with Fig. 1C, D; Table 1A with Table 1B). This result is indicative of the slow-rate of diffusion of EO from Gel-EOs that allows slow but constant diffusion of the oil from its system onto the bacteria prolonging its antibacterial effect on possibly slow growing bacteria such as *BS*. It is probable that the use of EO only was not able to withhold its antibacterial property at longer incubation time for *BS* but with the entrapment within the hydrogel network it could be substantially prolonged via sustained-release.

**Table 1:** ZOI of antibacterial assay of *B. subtilis* at various amounts; **A** 0.47 mg, 0.63 mg, 1.25 mg and 1.88 mg of *Zanthoxylum* oil applied in paper discs at incubation times 14 h and 22 h; and **B** at 1.25 mg and 1.88 mg of *Zanthoxylum* oil encapsulated in Fmoc-3F-Phe hydrogels at incubation times at 14 h and 22 h

A			
S. N.	Amount of *Zanthoxylum* used in paper disc	*B. subtilis*	
		14 h	22 h
1	Positive control	18 mm	15 mm
2	Solvent control		–
3	0.47 mg	9 ± 0.7 mm	–
4	0.63 mg	10 ± 0.7 mm	–
5	1.25 mg	10.5 ± 1.4 mm	–
6	1.88 mg	11 ± 0.5 mm	–

B			
S. N.	Amount of *Zanthoxylum* encapsulated in hydrogels	*B. subtilis*	
		14 h	22 h
1	Positive control	19 mm	17 mm
2	5 mM hydrogel	–	–
3	10% DMSO	–	–
4	15% DMSO	–	–
5	1.25 mg	10 ± 1 mm	9 ± 0.7 mm
6	1.88 mg	12 ± 1 mm	11.6 ± 1.4 mm

**Fig. 2:**
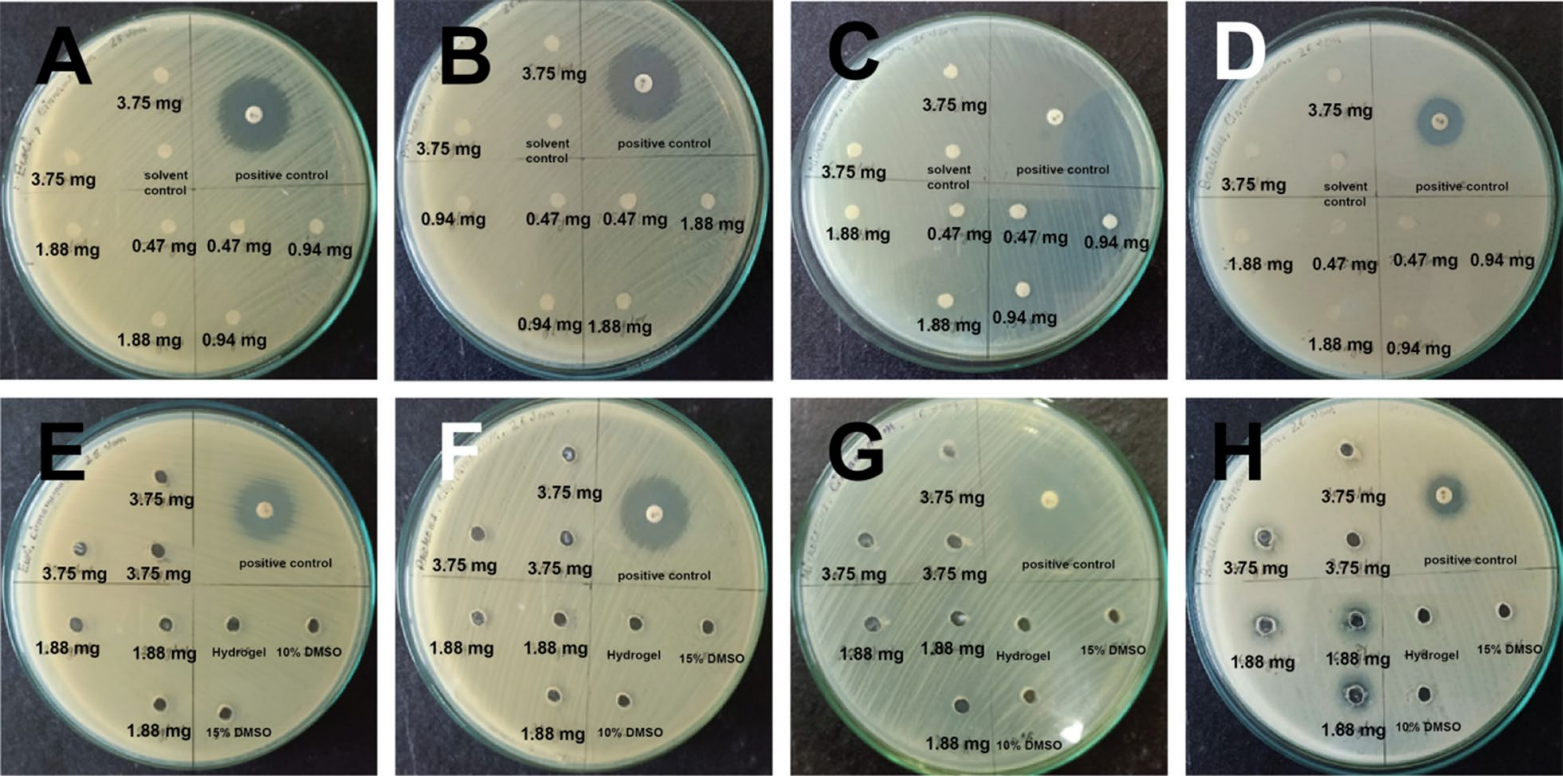
Antibacterial Assay of **A** *E. coli*, **B** *P. hauseri*, **C** *M. luteus* and **D** *B. subtilis* with various amounts (0.47 mg, 0.94 mg, 1.88 mg, 3.75 mg) of camphor oil directly applied in paper discs and **E** *E. coli*, **F** *P. hauseri*, **G** *M. luteus* and **H** *B. subtilis* with various amounts (1.88 mg and 3.75 mg) of camphor oil encapsulated in Fmoc-3F-Phe hydrogels at 14 h incubation time

With successful utilization of hydrogels for *Zanthoxylum* oil encapsulation for improved efficacy for antibacterial properties, we attempted further utilization of the gels for encapsulation of more volatile oil such as camphor oil. This oil in particularly is very difficult to handle owing to its subliming nature. The GC–MS done on this oil show chemical composition with 95% of *Cinnamomum*, which is known to be highly volatile (Additional file 1: Chromatogram 1, Table S3). Reports published in the past, show greatly varying results in its ability to inhibit various types of bacteria where some results show good effectivity against certain bacterial strains while other reports show case no such effectivity at all on the same types of strains [17–21]. While it may be that the oils isolated at varying geographical regions may attribute to such discrepancy, still its widely known that this oil has highly subliming nature which could lead to inconsistent results in repeated experiments. Therefore, in our experiments we attempted entrapping this volatile oil to form Gel-EOs and see if that may show improved results in bacterial inhibition. In our experiments, we found that we were unable to find any antibacterial effectivity by the oil itself when the oil was directly applied to the four bacterial assays using the disc method at various amounts of oil (0.47, 0.94, 1.88 and 3.75 mg) (Fig. 2A–D, Additional file 1: Table S2A). There was no observation even until 22 h of incubation time. However, when the experiment was repeated using Gel-EOs, much improved results were observed where the oil was able to display excellent antibacterial activity with clear ZOI against *ML* (9.3–13 mm) and *BS* (9.2–10 mm) at high amount of 1.88 and 3.75 mg of oil (compare Fig. 2C, D with G, H and Additional file 1: Table S2A with Table S2B). The ZOI remained substantial at even 22 h of incubation time with diminishing but still considerable ZOI (Additional file 1: Table S2B). It was interesting to observe that with the use the hydrogel, the bacterial inhibition could be obtained for *BS* and *MLs* bacteria while no inhibition could be obtained using only the oil. It can be hypothesized that due to volatile nature of the oil, fully exposed oil on the agar plate sublimed quickly not allowing it to be fully effective against the bacteria. However, with the Gel-EOs, the sublimation of the oil was constrained thus allowing the oil to act against the bacteria at longer period of time suggesting sustained-release modality of the active molecules from the gels to enhance antibacterial activity.

It should be noted that the concentrations of the hydrogelator, Fmoc-3F-Phe was kept at low concentration of 5 mM for all above experiments which show no antibacterial activity at this concentration although it does seem that it itself has antibacterial property at very high concentrations (Additional file 1: Fig. S2 and Table S5) [22]. However, it can be assured that the ZOI observed for both oils from Gel-EOs was obtained from EOs only because the Fmoc-3F-Phe solutions itself do not show any antibacterial property at this low concentrations (Additional file 1: Fig. S3 and Table S6).

Therefore, it appears that the use of hydrogel has ideal use case for slow growing bacteria where the slow-release mechanism of the EOs from the Gel-EOs allow prolonged potency against the bacteria. Since, *BS* is slow growing bacteria with doubling time of 120 min at 35 °C [23] in comparison to *EC* (25 min at 37 °C [24]), *PH* (28 min at 37C [25]) and *ML* (30 min at 30 °C [26]), it can be surmised from results above, that for both oils, Gel-EOs showed better results at longer time points. In case of *Zanthoxylum* Gel-EOs, the ZOI continued to persist even at 22 h while it had disappeared when oil was directly used. For camphor, the Gel-EOs showed clear ZOI at 22 h while the oil directly used showed none. Therefore, it is probable that when oil are directly used, slow sublimation of *Zanthoxylum* and complete sublimation of camphor oil does not allow it to be effective against slow growing bacteria such as *BS*. The slow-release mechanism of these oils from the hydrogel therefore favors effective activity against the *BS* bacteria at even prolonged time 14–22 h. Even in case of *ML*, the camphor Gel-EOs shows some activity against the bacteria with presence of distinct ZOI, while direct use fails to show any activity. This indicates that the camphor oil should actually be effective against *ML* and *BS* bacteria but the sublimation of the oil did not allow the oil to act against these bacteria. The direct capturing of the oils within the hydrogels and slow-releasing mechanism allowed the oil to work against these bacteria. It seems the oils is actually ineffective in prohibiting *EC* or *PH* growth in any case.

In conclusion, it appears that the volatile nature of the camphor oil was never really addressed in past, therefore it is possible that the volatile and subliming nature of the oil caused the reports from experiments previously to vary greatly and give inconsistent results. The encapsulation of such volatile oil in the hydrogel therefore allowed it to show antibacterial effect on *BS* and *ML* thus showcasing the application of lmw hydrogelator such as of Fmoc-3F-Phe to encapsulate volatile oils and thus enhance its antibacterial property via controlled-release of the oil from its system. Also, the encapsulation of antibacterial EOs seems particularly better to treat slow growing bacteria such as *BS*, where the encapsulation of the oils allows it to retain its effectivity even at longer times to work against bacteria that causes more harm at later times after exposure.

## Limitations

All the work was done with laboratory strains only. Experiments done with clinical strains would have showcased the oils applications in treating clinically relevant bacteria. However, since the work was done in a low-income country Nepal, working with clinical and pathological bacteria was not feasible due to its unavailability. Further, while the results above indicated better efficacy of the oils for slowing growing *BS*, future work needs to be done to test the efficacy of oils in varieties of slow growing bacteria.

## Supplementary Information

**Additional file 1.**

## Data Availability

The datasets supporting the conclusions of this article are included within the article and Additional file.

## Abbreviations

LMW: Low molecular weight
Fmoc-phe: Fluorenylmethoxycarbonyl-phenylalanine
GC–MS: Gas chromatography–mass spectroscopy
ZOI: Zone of inhibition
DMSO: Dimethyl sulfoxide
H_2_SO_4_: Sulfuric acid
BaSO_4_: Barium sulfate
