# Supplementary material for: Utilization of FMOC-3F-PHE hydrogel for encapsulation of Zanthoxylum armatum and Cinnamomum camphora oil for enhancing their antibacterial activity
Source: BMC Res Notes. 2022 Aug 12;15:278. doi: 10.1186/s13104-022-06163-4 (PMC9373397; doi:10.1186/s13104-022-06163-4)
Supplement: Supplementary file 1 — Additional file 1. [file 13104_2022_6163_MOESM1_ESM.docx]

**UTILIZATION OF FMOC-3F-PHE HYDROGEL FOR ENCAPSULATION OF *ZANTHOXYLUM ARMATUM* AND *CINNAMOMUM CAMPHORA* OIL FOR ENHANCING THEIR ANTIBACTERIAL ACTIVITY**

Nasla Shakya^1^, Santosh B.C^2^, Susan Joshi^2^, Annada Rajbhandary^1*^

^1^Research Institute for Bioscience and Biotechnology (RIBB), Kathmandu, Nepal

^2^Tri-Chandra multiple campus, Durbar Marga, Kathmandu, Nepal

## Additional file 1

Table of Contents

[Additional Information S2](#_Toc100950849)

[1.1 Materials S4](#_Toc100950850)

[1.2 Hydrodistillation S4](#_Toc100950851)

[1.3 GC-MS S4](#_Toc100950852)

[1.4 Hydrogel Preparation S6](#_Toc100950853)

[1.5 Specific Gravity of oils S7](#_Toc100950854)

[1.6 Bacterial culture and Agar Plate Preparation S7](#_Toc100950855)

[1.7 Antibacterial assay by disc diffusion method for *Zanthoxylum* essential oil S7](#_Toc100950856)

[1.8 Antibacterial assay by agar well method for *Cinnamomum camphora* essential oil S8](#_Toc100950857)

[1.9 Antibacterial assay by using essential oils incorporated in hydrogel on agar well method S8](#_Toc100950858)

**Figure S1:** Antibacterial Assay of **A)** *E. coli*, **B**) *P. hauseri*, **C**) *M. luteus* with various amounts (0.47 mg, 0.63 mg, 1.25 mg and 1.88 mg**)** of *zanthoxylum* oil applied in paper discs and **D**) *E. coli*, **E**) *P. hauseri*, **F**) *M. luteus* with various amounts (1.25 mg and 1.88 mg) of *zanthoxylum* oil encapsulated in Fmoc-3F-Phe hydrogels at 14 hrs incubation time……………………………………………………………………………………...S10

**Table S1**: ZOI of antibacterial assay of *E.coli*, *P. hauseri* and *M. luteus* at various amounts of *Zanthoxylum* oil **(** 0.47 mg, 0.63 mg, 1.25 mg and 1.88 mg ) applied in A) paper discs and with B) encapsulation in Fmoc-3F-Phe hydrogels at incubation times 14 hrs; Positive control: Chloraphenicol (C 30), Solvent control: Isopropanol, Negative control: Fmoc-3F-Phe with 2% DMSO; 10% and 15% DMSO………………………….S11

**Table S2**: ZOI of antibacterial assay of *E.coli*, *P. hauseri, M. luteus, B. subtilis* at various amounts of camphor oil **(**0.47 mg, 0.94 mg, 1.88 mg and 3.75 mg ) applied in A) paper discs and with B) encapsulation in Fmoc-3F-Phe hydrogels at incubation times 14 hrs and 22 hrs for comparison; Positive control: Chloraphenicol (C 30), Solvent control: Isopropanol, Negative control: Fmoc-3F-Phe with 2% DMSO; 10% and 15% DMSO…S12

**Chromatogram 1**: GC chromatogram and MS spectra of various chemical constituents of the *Cinnamomum camphora* leaves essential oil………………………………………... S13

**Table S3**: Chemical Composition of *Cinnamomum camphora* essential oil with percentage areas…………………………………………………………………………..S14

**Chromatogram 2**: GC chromatogram and MS spectra of various chemical constituents of the *Zanthoxylum armatum* seeds essential oil………………………………………..…..S15

**Table S4**: Chemical Composition of *Zanthoxylum armatum* essential oil with percentage areas………………………………………………………………………………………S16

**Figure S2:** Antibacterial Assay at 22 hrs of **A)** *M. luteus*, **B**) *B. subtilis* and **C**) *E. coli* at various concentrations (5 mM, 15 mM, 40 mM and 60 mM**)** of Fmoc-3F-Phe solution dissolved in DMSO applied in paper discs (after drying the solvent for 2 hrs) to observe antibacterial effects of Fmoc-3F-Phe hydrogelator itself…………………………….......S17

**Table S5**: ZOI of antibacterial assays at 22 hrs of *M. luteus*, *B. subtilis* and *E. coli* at various concentrations (5 mM, 15 mM, 40 mM and 60 mM**)** of Fmoc-3F-Phe solution dissolved in DMSO applied in paper discs (after drying the solvent for 2 hrs) to observe antibacterial effects of Fmoc-3F-Phe hydrogelator itself. Positive control: Chloraphenicol (C 30), Solvent control: DMSO…………….…………………………………………….S18

**Figure S3.** Antibacterial Assay at 22 hrs of **A)** *B. subtilis* and **B**) *E. coli* with 1.88 mg of *Zanthoxylum* oil and **C**) *B. subtilis* and D) *M. luteus* with 3.75 mg of camphor oil mixed with Fmoc-3F-Phe gelator in DMSO and applied in paper discs or with incorporation in Fmoc-3F-Phe hydrogel. Also, 5 mM of Fmoc-3F-Phe dissolved in DMSO has been applied in paper discs (after drying the solvent for 2 hrs) to observe antibacterial effects of Fmoc-3F-Phe hydrogelator itself incomparison to mixture of oils and gelator applied in paper discs and hydrogels incorporated with the oils…………………………………………..S19

**Figure S3.** Antibacterial Assay at 22 hrs of **A)** *B. subtilis* and **B**) *E. coli* with 1.88 mg of *Zanthoxylum* oil and **C**) *B. subtilis* and D) *M. luteus* with 3.75 mg of camphor oil mixed with Fmoc-3F-Phe gelator in DMSO and applied in paper discs or with incorporation in Fmoc-3F-Phe hydrogel. Also, 5 mM of Fmoc-3F-Phe dissolved in DMSO has been applied in paper discs (after drying the solvent for 2 hrs) to observe antibacterial effects of Fmoc-3F-Phe hydrogelator itself incomparison to mixture of oils and gelator applied in paper discs and hydrogels incorporated with the oils…………………………………………..S20

## 1.1 Materials

Fmoc-3F-Phe, DI water and organic solvents were purchased commercially and used without further purification. Bacterial strains - *Bacillus subtilis* ATCC66333, *Escherichia coli* ATCC 25922, *Micrococcus luteus* KACC 13377 and, *Proteus hauseri* NBRC 3851 were used. Seeds of Sichuan pepper were wild variety obtained from Chhedadad, ward no. 6 (Meduka), Jajarkot, Nepal. The leaves of *Cinnamomum Camphor* were obtained from Tribhuvan University, Kirtipur, Nepal. These were identified and authenticated from the Central Department of Botany, TU, Kirtipur, Kathmandu Nepal.

## 1.2 Hydrodistillation

The seeds of Sichuan pepper and leaves of *Cinnamomum camphor* were thoroughly cleaned, dried and extracted using Clevenger-type hydro-distillation apparatus. Amount of about 100 grams of sample (Sichuan pepper seed or camphor leaves) were placed in a round bottom flask. The flask were then filled with distilled water and fitted with Clevenger-type distillation apparatus as shown in Figure 3.1. The apparatus were heated using heating mantle at 100 °C for 4 hours followed by cooling at room temperature for 1 hour. The isolated essential oil of Sichuan pepper were dried using anhydrous sodium sulphate and stored for further uses. Camphor oil was isolated as it is.

## 1.3 GC-MS

Constituents of the *Zanthoxylum* and Camphor essential oil were analyzed by the GC-MS. Gas chromatography-mass spectrometry (GC-MS) analysis was carried out on Agilent Technologies with conditions as follows:

| **GC Programming** | |  |
| --- | --- | --- |
| **S. No.** | **Parameters** | **Values** |
| 1 | Column Oven Temperature | 50 °C |
| 2 | Injection Temperature | 250 °C |
| 3 | Carrier Gas | He |
| 4 | Primary Pressure | 300 - 500 Kpa |
| 5 | Flow control mode | Linear Velocity |
| 6 | Pressure | 53.8 Kpa |
| 7 | Total Flow | 112.3 mL/min |
| 8 | Column Flow | 1.35 mL/min |
| 9 | Linear Velocity | 29.8 cm/sec |
| 10 | Purge Flow | 3.0 mL/min |
| 11 | Split Ratio | 80 |

| **Column Oven Temperature Programming** | | |  |
| --- | --- | --- | --- |
|  | Rate (°C/min) | Final Temperature (°C) | Hold Time (Min.) |
| 0 | - | 50.0 | 1.00 |
| 1 | 3.00 | 230.0 | 9.00 |

Total Program Time = 70 min

**Column Specification of GCMS used by DPR**

| Name | Rtx-5MS |
| --- | --- |
| Length | 60.0 Meter |
| Thickness | 0.25 µm |
| Diameter | 0.32 mm |

| **MS Programming** |  |
| --- | --- |
| Ion Source Temperature | 250 °C |
| Interface Temperature | 200 °C |
| Solvent Cut Time | 5.5 min |
| Micro Scan Width | 0 u |
| Detector Voltage | Relative to the Tuning Result |
| Threshold | 0 |

Group #1 - Event #1

| S. N. | Start Time (Min.) | End Time (Min.) | Acq. Mode | Event Time (Sec) | Scan Speed | Start m/z | End m/z |
| --- | --- | --- | --- | --- | --- | --- | --- |
| 1 | 6.00 | 70.00 | Scan | 0.50 | 666 | 40.00 | 350.00 |
| 2 | 0.00 | 0.00 | Scan | 0.00 | 0.00 |  | 0.00 |
|  |  |  |  |  |  |  |  |

## 1.4 Hydrogel Preparation

Stock solution of 247 mM concentration of L-Fmoc-3-fluorophenyl-alanine was prepared by adding 10 mg of Fmoc-3F-phe to 100 µl of DMSO. Then the working concentration of 5 mM hydrogel was prepared by adding 490 µl of distilled water to 10 µl of hydrogel stock solution.

## 1.5 Specific Gravity of oils

Then the specific gravity of the *Zanthoxylum*and camphor essential oil was determined by measuring weight of 1 mL of essential oil. It was found to be 0.838gm/l for *Zanthoxylum armatum* oil and 0.850gm/l for *Cinnamomum camphora* oil.

## 1.6 Bacterial culture and Agar Plate Preparation

Overnight culture of gram positive bacteria *B. subtilis* and *M. luteus*and gram negative bacteria *E. coli and P. hauseri*were prepared in autoclaved Nutrient broth. The overnight cultures of bacteria were matched with 0.5 McFarland by diluting with additional nutrient broth solution. 0.5 McFarland was prepared by adding 0.1 ml of conc. H_2_SO_4_ in 9.9 ml of distilled water to make 1% H_2_SO_4_, 0.05 ml was discarded from 1% H_2_SO_4_and 0.05ml of 1% BaSO_4_ was added to it, then vortexed for 2 min. This was prepared in a clear culture bottle. Then Muller Hinton agar was prepared in distilled water, autoclaved and poured in the sterile petri plates of 14mm diameter. After the agar solidified, the bacteria were spread onto the agar with the help of the sterile Q-tips to cover entire surface of the agar. These agar plates were further utilized to either probe the ZOI of the essential oils using disc method or to investigate the release kinetics of the hydrogels with essential oils incorporated using well diffusion method.

## 1.7 Antibacterial assay by disc diffusion method for *Zanthoxylum* essential oil

Paper discs were prepared from the filter paper using punching machine then they were sterilized by autoclaving. Required concentration of as low as 0.47 mg of essential oils was prepared using isopropanol solvent for mixing the essential oil in the solvent. 10µl from the stock solution of the essential oil of required concentration was added to the paper disc on each side and the disc was allowed to air dry. Then the paper discs were placed on the agar plates with desired bacteria. The agar plates were then incubated at 37°C for 6 hrs and 22 hrs. Every tests were carried out in triplicates.

## 1.8 Antibacterial assay by agar well method for *Cinnamomum camphora* essential oil

Antibacterial assay for camphor essential oil could not utilized using disc method due to highly volatile nature of the oil. The oil sublimes as soon as placed on the discs. Therefore, well method was utilized to probe antibacterial assay of the oil. The agar wells with diameter of 6 mm were prepared using a sterile borer after spreading bacteria onto the agar plates. Since camphor essential oil is extremely volatile the essential oil container was kept on ice pack to avoid sublimation before transferring it to the wells. Then the required concentration of as high as 3.75 mg of essential oil dissolved in isopropanol was added to the wells and incubated for 6 to 22 hrs. Every tests were carried out in triplicates.

## 1.9 Antibacterial assay by using essential oils incorporated in hydrogel on agar well method

Agar plates with bacterial culture were prepared as described above and 6 mm wells were prepared using the sterile borer. The essential oils were incorporated in the hydrogels by adding the desired volume of essential oil in the Eppendorf tube. Then gelling solution prepared as described above in hydrogel preparation section was rapidly added to the oil and immediately transferred into the agar well plates. This allows hydrogels to form only in the wells of the agar plates rather than forming in the Eppendorf tubes. Then the plates were placed in the incubator at 37°C for 6 to 22 hrs. Camphor oil was kept on the ice pack before transferring adding it to the gels. Every tests were carried out in triplicates.

**
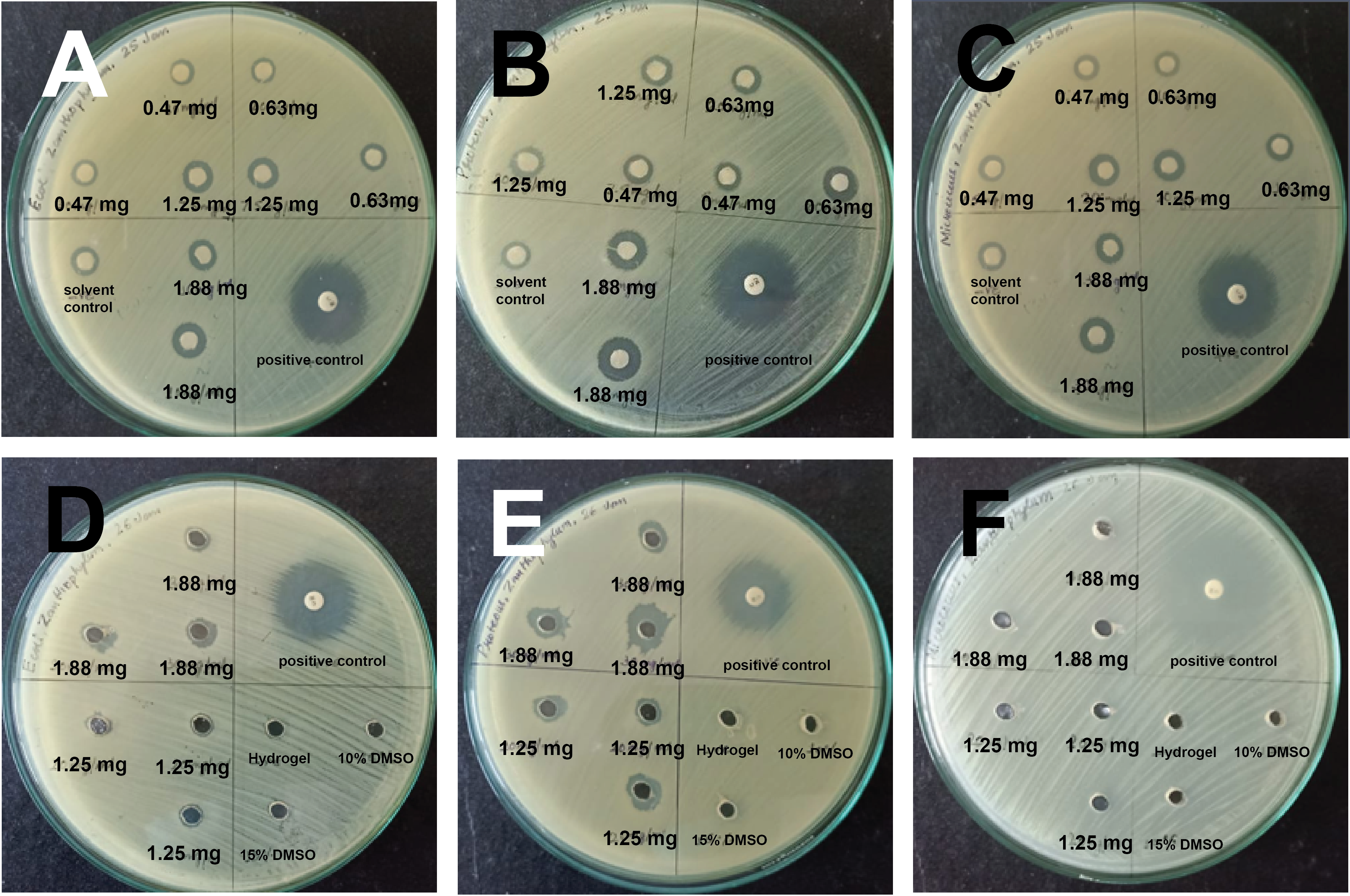
**

**Figure S1:** Antibacterial Assay of **A)** *E. coli*, **B**) *P. hauseri*, **C**) *M. luteus* with various amounts (0.47 mg, 0.63 mg, 1.25 mg and 1.88 mg**)** of *zanthoxylum* oil applied in paper discs and **D**) *E. coli*, **E**) *P. hauseri*, **F**) *M. luteus* with various amounts (1.25 mg and 1.88 mg) of *zanthoxylum* oil encapsulated in Fmoc-3F-Phe hydrogels at 14 hrs incubation time.

**A)**

| S. N. | Amount of *Zanthoxylum* used in paper disc | Bacteria used | | |
| --- | --- | --- | --- | --- |
|  |  | *E. coli* | *P. hauseri* | *M. luteus* |
| 1 | Positive Control | 25 mm | 27 mm | 25 mm |
| 2 | Solvent Control | 7 mm | 7 mm | 7 mm |
| 3 | 0.47 mg | 9 ±0.7 mm | 9±0.6 mm | 9±0.7 mm |
| 4 | 0.63 mg | 9.5±0.7mm | 9.5±0.7 mm | 10±0.7 mm |
| 5 | 1.25 mg | 10±0.6 mm | 12.5±0.7 mm | 13±1.4 mm |
| 6 | 1.88 mg | 11±0.8 mm | 12±1.2 mm | 15±0.8 mm |

**B)**

| S. N. | Amount of *Zanthoxylum*  encapsulated in hydrogels | Bacteria used | | |
| --- | --- | --- | --- | --- |
|  |  | *E. coli* | *P. hauseri* | *M. luteus* |
| 1 | Positive Control | 25 mm | 25 mm | 27 mm |
| 2 | 5 mm Hydrogel | - | - | - |
| 3 | 10 % DMSO | - | - | - |
| 4 | 15 % DMSO | - | - | - |
| 5 | 1.25 mg | 10.6±0.6 mm | 11.6±1.2 mm | 14±1.5 mm |
| 6 | 1.88 mg | 13±1 mm | 14.3±1.4 mm | 16±1 mm |

**Table S1**: ZOI of antibacterial assay of *E.coli*, *P. hauseri* and *M. luteus* at various amounts of *Zanthoxylum* oil (0.47 mg, 0.63 mg, 1.25 mg and 1.88 mg ) applied in A) paper discs and with B) encapsulation in Fmoc-3F-Phe hydrogels at incubation times 14 hrs; Positive control: Chloraphenicol (C 30), Solvent control: Isopropanol, Negative control: Fmoc-3F-Phe with 2% DMSO; 10% and 15% DMSO

**A)**

| S. N. | Amount of camphor oil used in paper disc | Bacteria used | | | |
| --- | --- | --- | --- | --- | --- |
|  |  | *E. coli* | *P. hauseri* | *M. luteus* | *B. subtilis* |
| 1 | Positive Control | 27 mm | 25 mm | 30 mm | 18 mm |
| 2 | Solvent Control | - | - | - | - |
| 3 | 0.47 mg | - | - | - | - |
| 4 | 0.94 mg | - | - | - | - |
| 5 | 1.88 mg | - | - | - | - |
| 6 | 3.75 mg | - | - | - | - |

**B)**

| S. N. | Amount of camphor oil encapsulated in hydrogels | Bacteria used | | | | | |
| --- | --- | --- | --- | --- | --- | --- | --- |
|  |  | *E. coli* | *P.hauseri* | *M. luteus* | | *B. subtilis* | |
|  |  | 14 hrs | 14 hrs | 14 hrs | 22 hrs | 14 hrs | 22hrs |
| 1 | Positive Control | 28 mm | 25 mm | 29 mm | 27 mm | 19 mm | 15mm |
| 2 | 5 mM Hydrogel | - | - | - | - |  |  |
| 3 | 10 % DMSO | - | - | - | - |  |  |
| 4 | 15 % DMSO | - | - | - | - |  |  |
| 5 | 1.88 mg | - | - | 11±0.6 mm | 9.3±1 mm | 10±1 mm | 9.2±0.6 mm |
| 6 | 3.75 mg | - | - | 13±0.6 mm | 12.3±0.6 mm | 12±1.8 mm | 10±1 mm |

**Table S2**: ZOI of antibacterial assay of *E.coli*, *P. hauseri, M. luteus, B. subtilis* at various amounts of camphor oil **(**0.47 mg, 0.94 mg, 1.88 mg and 3.75 mg ) applied in A) paper discs and with B) encapsulation in Fmoc-3F-Phe hydrogels at incubation times 14 hrs and 22 hrs for comparison; Positive control: Chloraphenicol (C 30), Solvent control: Isopropanol, Negative control: Fmoc-3F-Phe with 2% DMSO; 10% and 15% DMSO


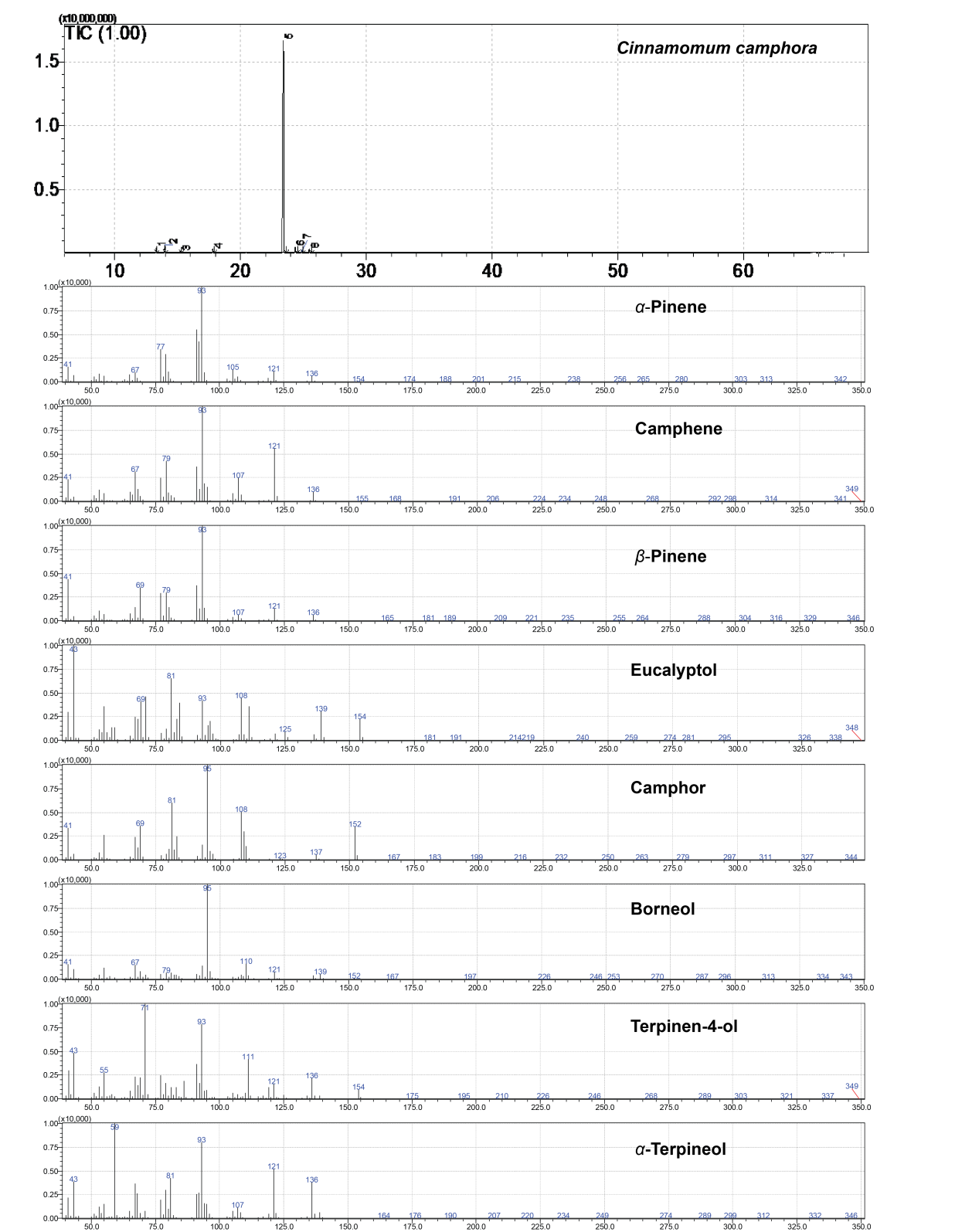


**Chromatogram 1**: GC chromatogram and MS spectra of various chemical constituents of the *Cinnamomum camphora* leaves essential oil.

| S. N. | Name of the compounds | Retention time | Percentage Area |
| --- | --- | --- | --- |
| 1 | *α*-Pinene | 13.269 | 1.34 |
| 2 | Camphene | 13.954 | 2.09 |
| 3 | *β*-Pinene | 15.221 | 0.53 |
| 4 | Eculyptol | 17.779 | 1.06 |
| 5 | Camphor | 23.407 | 91.39 |
| 6 | Borneol | 24.343 | 1.82 |
| 7 | Terpinen-4-ol | 24.853 | 0.56 |
| 8 | *α*-Terpineol | 25.461 | 1.20 |

**Table S3**: Chemical Composition of *Cinnamomum camphora* essential oil with percentage areas.

#
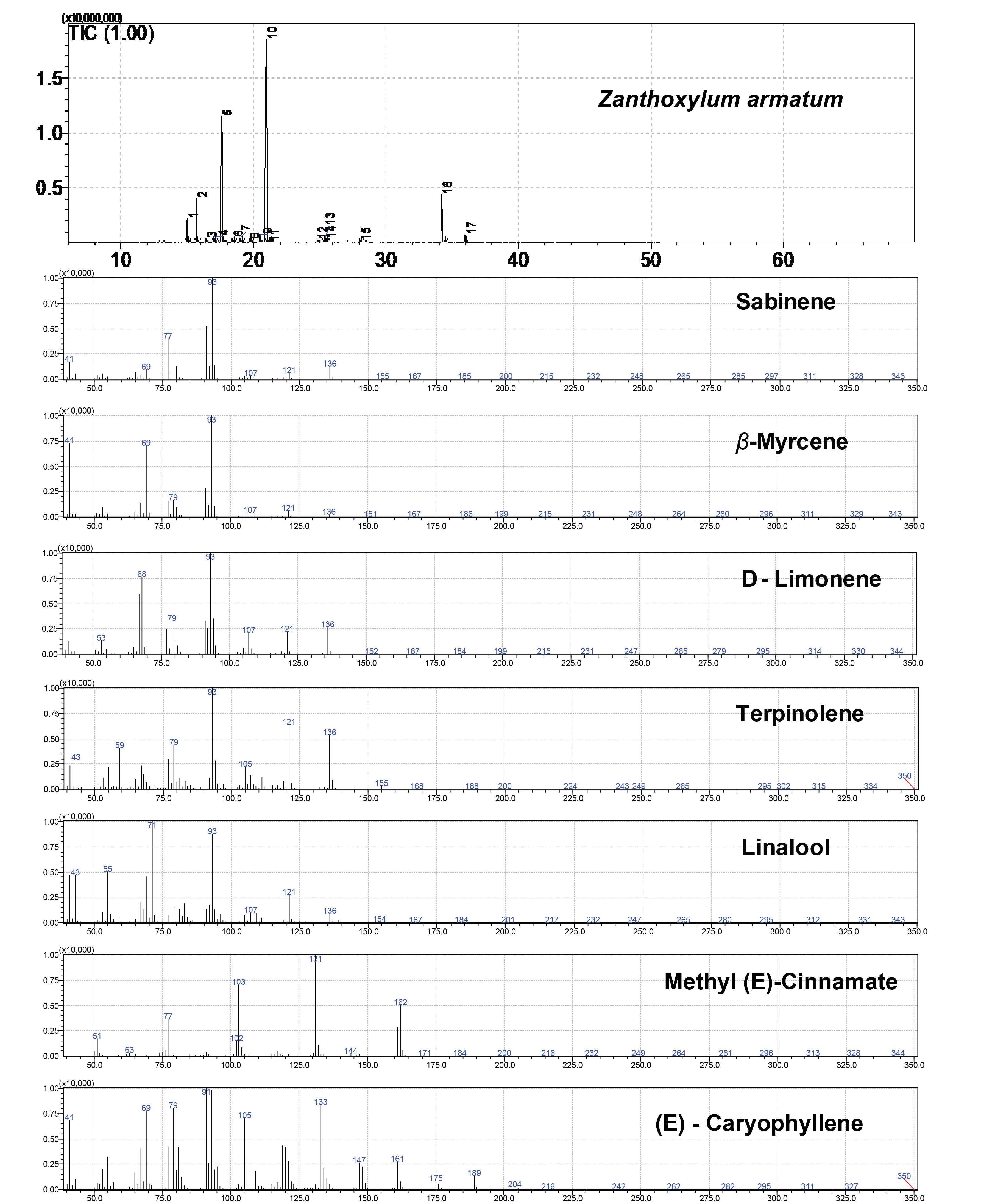


**Chromatogram 2**: GC chromatogram and MS spectra of various chemical constituents of the *Zanthoxylum armatum* seeds essential oil.

| S. N. | Name of the compounds | Retention time | Percentage Area |
| --- | --- | --- | --- |
| 1 | Sabinene | 14.996 | 2.86 |
| 2 | Myrcene | 15.686 | 5.26 |
| 3 | *α*-Phellandrene | 16.410 | 0.53 |
| 4 | *α*-Terpinene | 16.981 | 0.72 |
| 5 | D-Limonene | 17.589 | 28.60 |
| 6 | *β*-Ocimene | 18.395 | 0.62 |
| 7 | *γ*-Terpinene | 18.999 | 0.59 |
| 8 | Trans-Linalooloxide | 19.686 | 0.40 |
| 9 | Terpinolene | 20.452 | 1.14 |
| 10 | Linalool | 20.988 | 48.85 |
| 11 | Nonanal | 21.136 | 0.50 |
| 12 | Terpinen-4-ol | 24.822 | 0.56 |
| 13 | Cryptone | 25.316 | 0.46 |
| 14 | *α*-Terpineol | 25.435 | 0.62 |
| 15 | 4-Hexen-1-ol, 5-methyl-2-(1-methylethenyl)- | 28.042 | 0.38 |
| 16 | Methyl-Cinnamate | 34.236 | 6.88 |
| 17 | Caryophyllene | 35.990 | 1.07 |

**Table S4**: Chemical Composition of *Zanthoxylum armatum* essential oil with percentage areas.

**
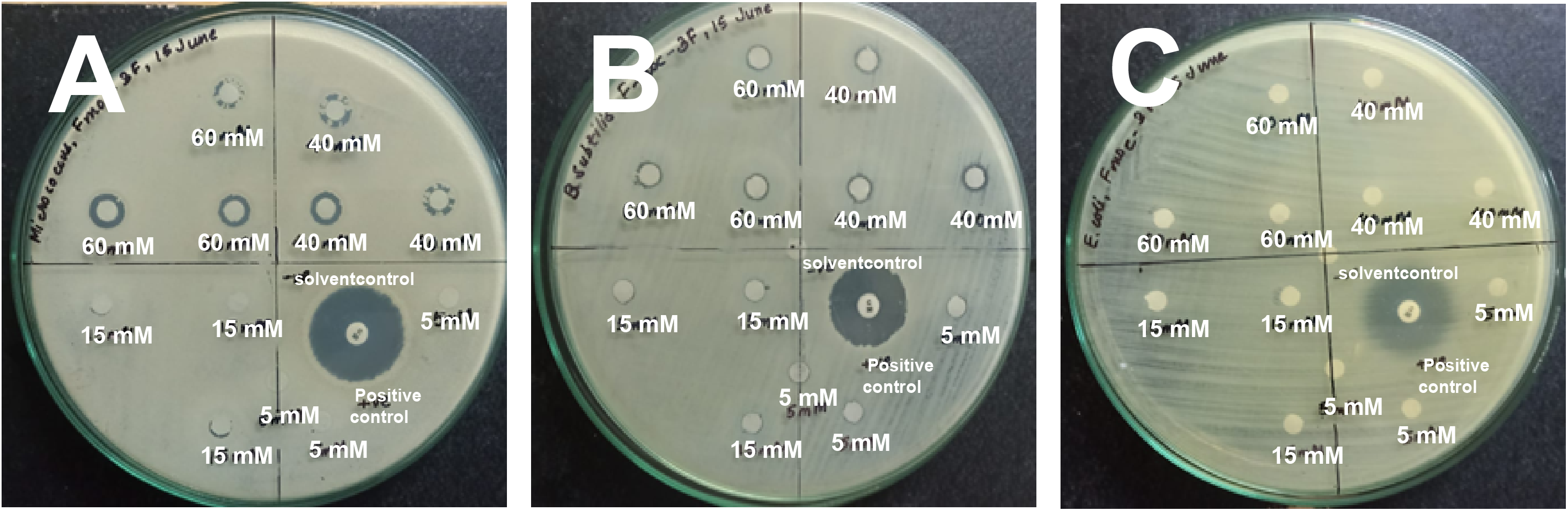
Figure S2:** Antibacterial Assay at 22 hrs of **A)** *M. luteus*, **B**) *B. subtilis* and **C**) *E. coli* at various concentrations (5 mM, 15 mM, 40 mM and 60 mM**)** of Fmoc-3F-Phe solution dissolved in DMSO applied in paper discs (after drying the solvent for 2 hrs) to observe antibacterial effects of Fmoc-3F-Phe hydrogelator itself. Low concentrations of the hydrogelator, Fmoc-3F-Phe at 5 mM and 15 mM showed no antibacterial activity. Only at very high concentrations of the hydrogelator (40 mM and 60 mM), any antibacterial activity is seen for only gram positive bacteria.

| S. N. | Concentration of Fmoc-3F-Phe used | Bacteria used | | |
| --- | --- | --- | --- | --- |
|  |  | *M. luteus* | *B. subtilis* | *E. coli* |
| 1 | Positive Control | 27 mm | 23 mm | 23 mm |
| 2 | Solvent Control (DMSO) | - | - | - |
| 3 | 5 mM | - | - | - |
| 4 | 15 mM | - | - | - |
| 5 | 40 mM | 8.7±0.6 mm | 7.6±0.6 mm | - |
| 6 | 60 mM | 9.3±1.2 mm | 7.5±0.6 mm | - |

**Table S5**: ZOI of antibacterial assays at 22 hrs of *M. luteus*, *B. subtilis* and *E. coli* at various concentrations (5 mM, 15 mM, 40 mM and 60 mM**)** of Fmoc-3F-Phe solution dissolved in DMSO applied in paper discs (after drying the solvent for 2 hrs) to observe antibacterial effects of Fmoc-3F-Phe hydrogelator itself. Positive control: Chloraphenicol (C 30), Solvent control: DMSO.


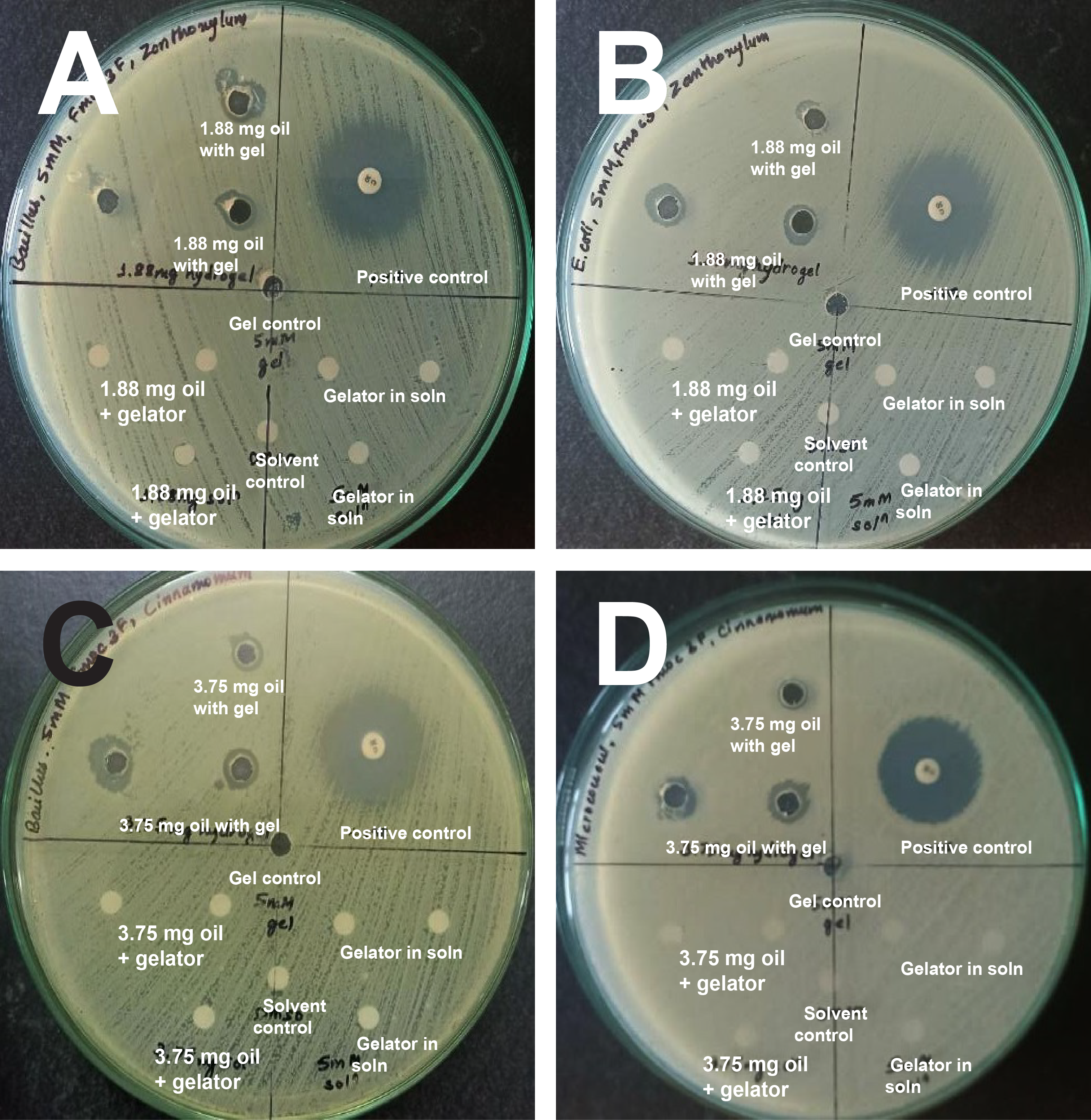


**Figure S3.** Antibacterial Assay at 22 hrs of **A)** *B. subtilis* and **B**) *E. coli* with 1.88 mg of *Zanthoxylum* oil and **C**) *B. subtilis* and D) *M. luteus* with 3.75 mg of camphor oil mixed with Fmoc-3F-Phe gelator in DMSO and applied in paper discs or with incorporation in Fmoc-3F-Phe hydrogel. Also, 5 mM of Fmoc-3F-Phe dissolved in DMSO has been applied in paper discs (after drying the solvent for 2 hrs) to observe antibacterial effects of Fmoc-3F-Phe hydrogelator itself incomparison to mixture of oils and gelator applied in paper discs and hydrogels incorporated with the oils. It can be observed that only hydrogels with zanthoxylum oil or camphor oil show any ZOI. Fmoc-3F-Phe in DMSO solution show no ZOI at low concentration of 5 mM, while oil and Fmoc-3F-Phe gelator dissolved in DMSO also showed no ZOI due to sublimation of the essential oils after drying of the DMSO for 2 hrs on the paper discs.

| S. N. | Substrate used | Bacteria used | | | |
| --- | --- | --- | --- | --- | --- |
|  |  | *B. subtilis*  1.88 mg *Zanthoxylum* | *E.coli*  1.88 mg  *Zanthoxylum* | *B. subtilis*  3.75 mg  Camphor | *M. luteus*  3.75 mg  Camphor |
| 1 | Positive Control | 22 mm | 25 mm | 23 mm | 26 mm |
| 2 | Solvent Control | - | - | - | - |
| 3 | 5mM Hydrogel with oil | 10±1.4 mm | 10.5±0.7 mm | 9.7±2.1 mm | 8.5±0.8 mm |
| 4 | Hydrogel Control (5 mM) | - | - | - | - |
| 5 | Fmoc-3F-Phe in solution of DMSO (5 mM) | - | - | - | - |
| 6 | Mixture of oils with gelator in DMSO | - | - | - | - |

**Table S6**: ZOI of antibacterial Assay at 22 hrs of **A)** *B. subtilis* and **B**) *E. coli* with 1.88 mg of *Zanthoxylum* oil and **C**) *B. subtilis* and D) *M. luteus* with 3.75 mg of camphor oil mixed with Fmoc-3F-Phe gelator in DMSO and applied in paper discs or with incorporation in Fmoc-3F-Phe hydrogel. Also, 5 mM of Fmoc-3F-Phe dissolved in DMSO has been applied in paper discs (after drying the solvent for 2 hrs) to observe antibacterial effects of Fmoc-3F-Phe hydrogelator itself incomparison to mixture of oils and gelator incorporated with the oils. Positive control: Chloraphenicol (C 30), Negative control: 5 mM Fmoc-3F-Phe hydrogel, Solvent control: DMSO.
